# The role of complement C1q in atrial fibrillation: a marker for disease progression and surgical outcomes

**DOI:** 10.3389/fcvm.2025.1512187

**Published:** 2025-05-02

**Authors:** Yuefeng Ju, MaoJing Wang, Yang Ji, Zhihui Wang, Wenzhuo Wang, Feiyue Liu, Qing Zhao

**Affiliations:** Department of Cardiology, The Affiliated Hospital of Qingdao University, Qingdao, China

**Keywords:** atrial fibrillation, plasma complement C1q, myocardial fibrosis, complex fractionated atrial electrograms, pulmonary vein isolation

## Abstract

**Background and objective:**

The complement system plays a crucial role in the pathogenesis and progression of cardiovascular diseases. C1q, a key initiator of the classical pathway, is closely associated with various chronic inflammatory conditions. This observational study aims to elucidate the potential risk relationship between serum complement C1q levels and atrial fibrillation (AF).

**Materials and methods:**

This retrospective cohort study included 812 AF patients treated at the Affiliated Hospital of Qingdao University from January 2020 to October 2022, comprising 694 patients in the paroxysmal AF group and 118 in the persistent AF group. Serum complement C1q levels were measured using an enzyme-linked immunosorbent assay (ELISA).

**Results:**

Serum C1q levels in the AF group were significantly lower than those in the control group (*P* < 0.001). Logistic regression analysis indicated that reduced plasma C1q levels were independently associated with the incidence of AF (95% CI = 0.974–0.981, *P* = 0.001). Additionally, ROC curve analysis confirmed the close association between plasma C1q levels and AF, highlighting the predictive value of C1q for AF. Further investigation revealed that C1q serves as an independent risk factor for complex fractionated atrial electrograms (CFAE) in the superior left atrium of paroxysmal AF patients (95% CI = 0.984–0.998, *P* = 0.031), suggesting its potential as a clinical indicator for guiding AF surgical interventions.

**Conclusion:**

Serum C1q levels are significantly reduced in patients with AF. The presence of CFAE in the superior left atrium of paroxysmal AF patients may be potentially associated with C1q levels. Low complement levels are associated with atrial fibrillation compared to individuals without AF and may represent a potential underlying cause of impaired sinus rhythm maintenance following pulmonary vein isolation. Complement C1q may play a critical role in the pathogenesis of AF.

## 1 Introduction

Atrial fibrillation is widely recognized as one of the leading causes of disability and mortality worldwide (1). However, the molecular mechanisms underlying atrial fibrillation (AF) remain incompletely characterized, resulting in persistent risks of heart failure, thromboembolic events, stroke, and even ventricular fibrillation (VF) induction in AF patients (2). Myocardial fibrosis induces the generation of atrial ectopic potentials, which are key contributors to the development of AF (3). Studies have demonstrated that the complement system exacerbates fibrotic processes in cardiomyocytes and vascular endothelial cells by triggering inflammatory responses, and it has been found to be closely associated with cardiovascular diseases such as coronary artery disease and aortic stenosis (4–7).

As a recognition molecule of the classical complement pathway, C1q binds to immune cells activated by antigens, initiating the classical pathway and regulating adaptive immunity (5, 6, 8, 9), Furthermore, C1q can activate non-classical pathways, such as Wnt signaling, through protease-cleaved alternative targets (5, 10–16). More importantly, C1q protects intracellular molecules and prevents the production of autoantibodies (17). Our observations revealed that serum C1q levels are generally lower in AF patients. Reduced synthesis, increased consumption, and depletion due to immune complexes or anti-C1q autoantibodies (HUVS) were closely associated with decreased C1q levels. Therefore, this study aims to uncover the potential relationship between complement C1q and AF and to explore the underlying mechanisms linking C1q to AF.

## 2 Subjects and methods

### 2.1 Subjects

**Figure 1:**
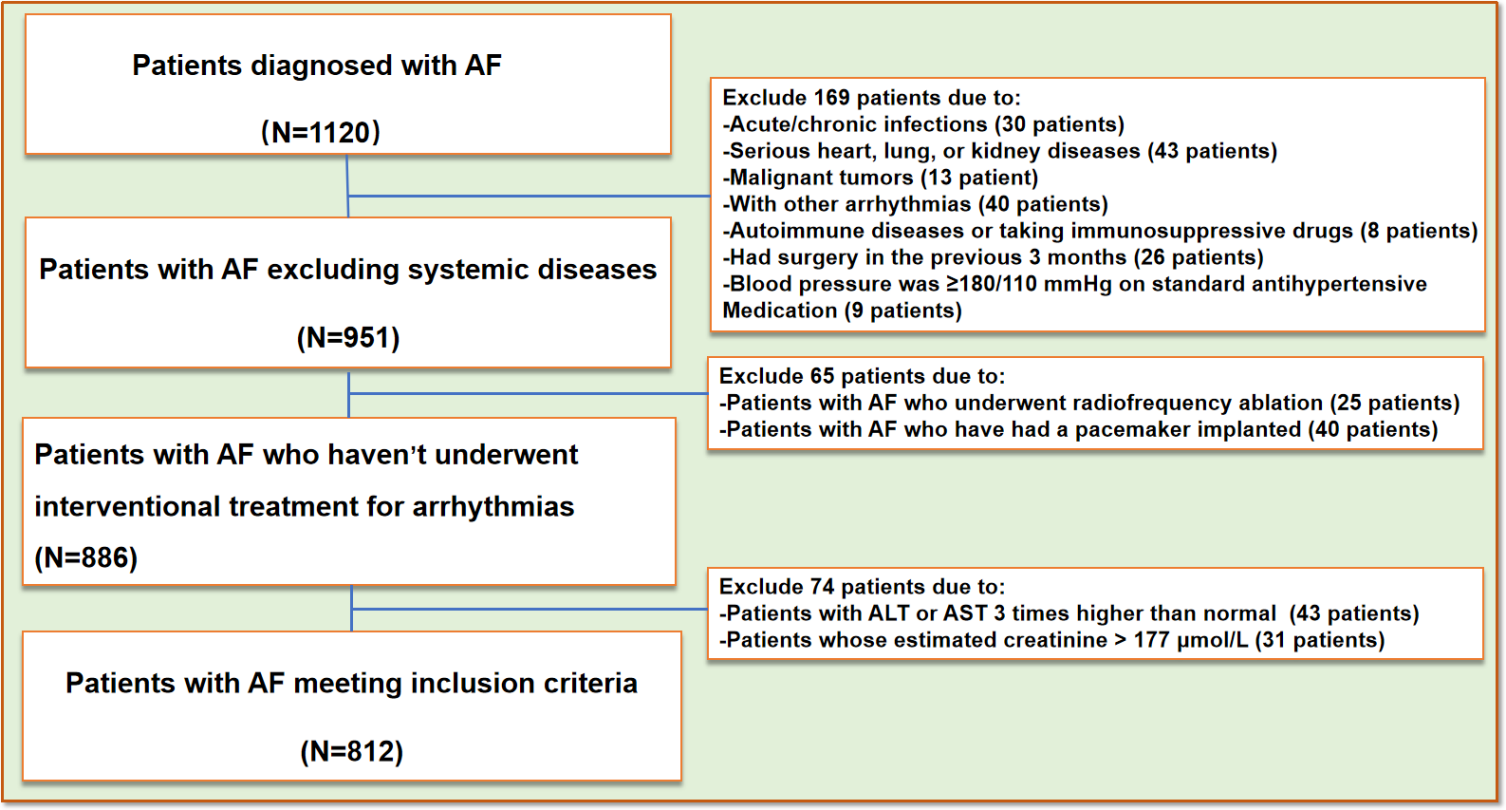
Flow-chart. AF, atrial fibrillation; AST, aspartate aminotransferase; ALT, alanine aminotransferase.

We conducted a retrospective study by collecting clinical data from patients treated at the Affiliated Hospital of Qingdao University between January 2020 and October 2022. A total of 812 patients (aged ≥ 18 years) clinically diagnosed with AF were included in the AF group, which was further subdivided into two subgroups: the persistent AF group (*n* = 118) and the paroxysmal AF group (*n* = 694). The control group comprised 1,012 non-AF participants recruited from the same health examination cohort at our institution during the study period. All enrolled participants underwent a rigorous screening protocol to exclude individuals with conditions potentially affecting serum complement C1q levels (Figure 1). Classification was performed according to the 2014 AHA/ACC/HRS AF management guidelines (18), Patients with AF episodes lasting more than 7 days without spontaneous termination or requiring pharmacological or electrical cardioversion were classified as part of the persistent AF group. Conversely, self-limiting AF episodes lasting less than 7 days were classified as part of the paroxysmal AF group. Exclusion criteria included patients with AF episodes related to other arrhythmias, those with pacemaker-induced AF, patients meeting the diagnostic criteria for acute coronary syndrome or non-stenotic acute myocardial infarction, patients with blood pressure ≥180/110 mmHg despite standard antihypertensive treatment, those with congestive heart failure (NYHA class III or IV), dilated or hypertrophic cardiomyopathy, valvular heart disease, or congenital heart disease. Patients experiencing acute cerebrovascular events or those who had undergone surgery within the last 3 months were also excluded. Additional exclusion criteria included patients with ALT or AST levels three times higher than the normal range, an estimated glomerular filtration rate (eGFR) < 15 ml/(min·1.73 m²), or creatinine >115 *μ*mol/L. Patients with malignancies, acute or chronic infections, and autoimmune diseases (8, 19) were also excluded. The study was approved by the institutional review board, and informed consent was obtained from all participants.

### 2.2 Methods

Peripheral blood samples of patients and controls were collected at admission into study. Within 30 min, blood samples were centrifuged at 3,000 g for 10 min, and afterwards plasma samples were stored at −80°C for final analysis. Plasma C1q levels were quantitatively measured utilizing the enzyme-linked immunosorbent assay with a commercially available kit according to the manufacturer's instructions.

All patients were staged using the 2014 AHA/ACC/HRS Guidelines for the Management of Patients with Atrial Fibrillation (18) as the basis for the diagnostic staging of atrial fibrillation, and patients in sinus rhythm were used as controls.

### 2.3 Echocardiography

Two-dimensional transthoracic echocardiography was performed using GE Vivid E9 as the same with a 3.5 MHz transducer. Transthoracic echocardiography was completed by experienced doctors on all subjects to evaluate the characteristics of their left atrial (LA), and left ventricular ejection fraction (LVEF) at the time of admission.

### 2.4 AF assessment

All participants were differentiated between sinus rhythm and AF rhythm through 12-lead electrocardiogram (ECG)/72 h Holter monitoring, medical history, and clinical evaluation, with further classification into persistent and paroxysmal AF subtypes.

### 2.5 Pulmonary vein isolation procedure

**Figure 2:**
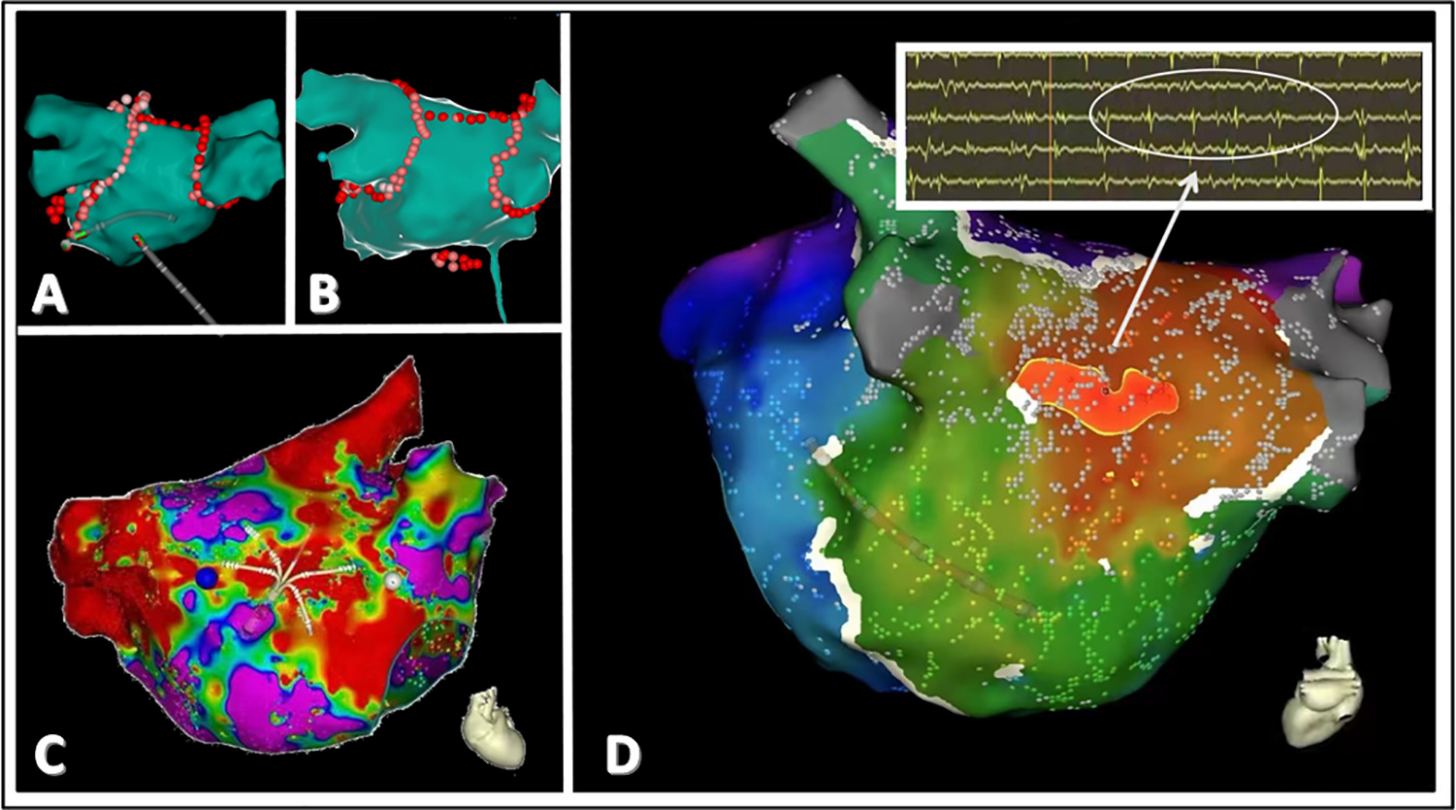
A schematic representation of CFAE in patients with paroxysmal atrial fibrillation. **(A,B)** depict the classic pulmonary vein isolation ablation and roofline ablation treatments. **(C)** demonstrates the localization of abnormal potential origins. **(D)** highlights the origin of abnormal potentials in the left atrium, with red indicating the site of the earliest myocardial potential. Electrophysiological testing reveals fractionated and fragmented potentials at this location, characteristic of CFAE.

Intracardiac electrograms were recorded using an advanced electrophysiological system. Three-dimensional electroanatomic mapping was conducted using a pulmonary vein mapping catheter. Paroxysmal AF patients with complex fractionated atrial electrograms (CFAE) were identified and recorded during the surgical procedure. CFAE identification strictly adhered to the Nademanee criteria: (1) electrogram amplitude <0.15 mV, (2) ≥3 deflections per 50 ms window, and (3) continuous electrical activity persisting >70% of AF cycle length. Automated quantification was performed using CARTO® CFAE software (v7.0, Biosense Webster) with thresholds set as: CFAE-mean ≤ 120 ms, SCI ≤ 60 ms, and ICL ≥ 6. All CFAE sites were validated by offline review of unannotated electrograms by two independent electrophysiologists. Systemic anticoagulation was achieved with intravenous heparin during the procedure to maintain an activated clotting time of 350–400 s. An open-irrigated tip catheter was used. Following the completion of protocol-based ablation, the procedure was concluded when no AF recurrence was observed for 10 min after isoproterenol infusion (5–20 μg/min, depending on the use of β-blockers, targeting a sinus heart rate of 120 bpm) (Figure 2).

### 2.6 Statistical analysis

Normally distributed data (continuous variables) were expressed as mean ± standard deviation. Non-normally distributed data were expressed as median of interquartile range or interquartile spacing, with frequencies and percentages indicating the characteristics of the categorical variables, Comparisons between groups of normally distributed data were made using unpaired *t*-tests. Non-parametric tests were used for comparison of non-normally distributed data between groups. Comparisons of categorical variables were analyzed using the chi-square test or Fisher's exact test. Binary logistic regression models were used for multivariate analysis. The Pearson correlation test was used to calculate the correlation between normally distributed data, and the Spearman correlation test was used to analyze the correlation between non-normally distributed data. All statistical analyses were performed using a commercial statistical program (SPSS) to analyze data. Two-sided *p* values <0.05 were considered statistically significant differences.

### 2.7 Ethics statement

This study was performed in line with the principles of the Declaration of Helsinki. Approval was granted by the Ethics Committee of the Affiliated Hospital of Qingdao University (QYFY-WZLL-28406).

## 3 Results

### 3.1 Clinical characteristics

A total of 1,120 AF participants were recruited. After a rigorous screening process (Figure 1), 812 AF participants were ultimately included in our study and classified into two groups based on their clinical presentation: the persistent AF group and the paroxysmal AF group. A control group consisting of 1,012 age- and sex-matched non-AF participants was also included. No statistically significant differences (*p* > 0.05) were observed between AF subjects and non-AF controls in demographic and anthropometric parameters, including gender, age, or body mass index (BMI). Tables 1–3 summarize the clinical characteristics, laboratory findings, and imaging results of the three groups. Of the 812 AF patients, the mean age was 55.88 ± 11.31 years, and the mean BMI was 26.15 ± 3.30 kg/m², with a total of 268 (33%) being female. Among the AF patients, 118 (14.5%) were classified as having persistent AF, while 694 (85.5%) were classified as having paroxysmal AF. Of the AF patients, 701 underwent catheter-based radiofrequency ablation, of which 600 (85.6%) were diagnosed with paroxysmal AF.

**Table 1:** Clinical characteristics of patients (*n* = 1,824).

Parameters	Non-AF Controls	AF Subjects	*P*-value
	(*n* = 1,012)	(*n* = 812)	
Female *n* (%)	489 (48.3)	268 (33.0)	0.479
Age(years)	54.87 ± 10.65	55.88 ± 11.31	0.085
SBP (mmHg)	129.22 ± 16.23	131.95 ± 16.02	0.326
DBP (mmHg)	79.13 ± 11.57	79.58 ± 11.42	0.402
BMI (kg/m^2^)	25.29 ± 3.26	26.15 ± 3.30	0.368
Smoking	210 (20.8)	178 (21.9)	0.195
Drinking	248 (24.5)	209 (25.7)	0.012
Hypertension	382 (37.8)	359 (44.2)	0.005
Diabetes	207 (20.5)	106 (33.9)	0.410
CHA₂DS₂-VASc Score	-	3.45 ± 1.74	-
Laboratory examination			
C1q(mg/L)	178.89 ± 30.31	160.93 ± 33.10	<0.001
Cr (μmol/L)	76.63 ± 13.07	75.42 ± 17.31	0.099
D-Dimer (μg/L)	308.54 ± 122.36	298.49 ± 131.55	0.092
FIB (g/L)	2.71 ± 0.53	2.75 ± 0.54	0.095
Echocardiography			
LA enlargement	501 (49.5)	635 (78.20)	<0.001
LVEF (%)	62.88 ± 3.96	58.94 ± 5.35	<0.001

SBP, systolic blood pressure; DBP, diastolic blood pressure; BMI, body mass index; Cr, creatinine; FIB, fibrinogen; LA, left atial; LA enlargement: High left atrium enlargement is defined as: left atrial area ≥20 cm^2^ or left atrial diameter >40 mm; LVEF, left ventricular ejection fraction.

**Table 2:** The levels of complement C1q in patients.

Parameters	The controls	AF		
	(*n* = 1,012)	Paroxysmal AF patients	Persistent AF patients	Total
		(*n* = 694)	(*n* = 118)	(*n* = 812)
C1q(mg/L)	178.89 ± 30.31	159.67 ± 32.71	155.03 ± 34.03	159.00 ± 32.92

**Table 3:** Echocardiography characteristics of patients.

Parameters	The controls	AF		
	(*n* = 1,012)	Paroxysmal AF patients	Persistent AF patients	Total
		(*n* = 694)	(*n* = 118)	(*n* = 812)
LAD				
35–40 mm	721 (71.24)	74 (10.66)	19 (16.10)	93 (11.46)
40–45 mm	100 (10.12)	121 (17.44)	14 (11.86)	134 (16.50)
46–55 mm	171 (16.90)	302 (43.52)	53 (44.92)	355 (43.72)
≥55 mm	20 (1.98)	114 (16.43)	19 (16.10)	133 (16.38)

LAD, left atrial diameter.

### 3.2 Atrial fibrillation

**Figure 3:**
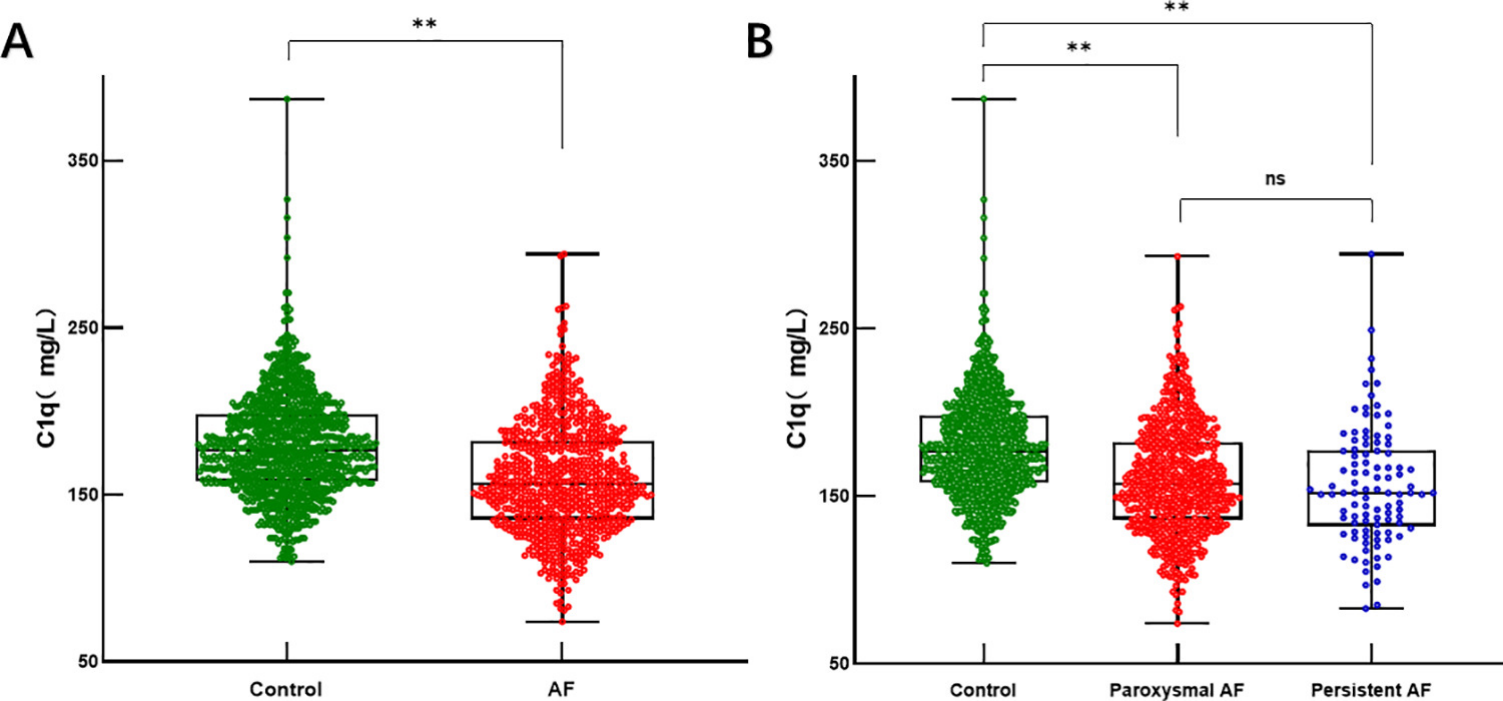
Serum C1q levels in AF and control groups. **(A)** Box and scatterplot of serum C1q levels in patients of Control, AF groups; **(B)** Box and scatterplot of serum C1q levels in patients of Control, paroxysmal AF, persistent AF groups. The horizontal line in the middle, top and bottom indicates the median value, the 75th and 25th percentiles, respectively; the dots represent each individual. *P < 0.05, **P < 0.01. AF, atrial fibrillation; C1q, complement component 1q.

Compared with the non-AF control group, serum C1q levels were significantly decreased in AF participants (AF group: 159.00 ± 32.92 mg/L vs. control group: 178.89 ± 30.31 mg/L, *P* < 0.001) (Table 1, Figure 3A), with even lower levels observed in the persistent AF subgroup (persistent AF group: 155.03 ± 34.03 mg/L vs. control group: 178.89 ± 30.31 mg/L, *P* < 0.001) (Table 2, Figure 3B). To evaluate whether complement C1q is an independent risk factor for AF, we included clinical and laboratory characteristics such as hypertension, diabetes, BMI, smoking, systolic blood pressure, alcohol consumption, serum creatinine levels, D-dimer, and left ventricular ejection fraction (LVEF) in a logistic regression analysis. After adjusting for confounding factors related to the incidence of AF, we found that C1q (95% CI = 0.974–0.980, *P* = 0.001), LVEF (95% CI = 0.766–1.814, *P* < 0.001), and hypertension (95% CI = 1.024–1.580, *P* = 0.030) were independent risk factors associated with AF. Among them, plasma C1q concentration was negatively correlated with AF (Table 4).

**Table 4:** Univariate and multivariable logistic regression analyses.

Parameters	Univariate analysis		Multivariable analysis	
	95% CI	*P*	95% CI	*P*
Complement C1q	0.976–0.983	<0.001	0.974–0.981	0.001*
Hypertension	1.083–1.577	0.005	1.024–1.580	0.030*
Diabetes	0.752–1.193	0.644		
BMI	0.996–1.010	0.385		
SBP	0.997–1.009	0.326		
Drinking	1.062–1.645	0.012	0.903–1.497	0.243
Smoking	0.694–1.077	0.195		
Cr	0.989–1.001	0.089		
D-dimer	0.999–1.000	0.093		
LVEF	0.775–0.821	<0.001	0.766–0.814	<0.001*

BMI, body mass index; SBP, systolic blood pressure; Cr, creatinine; LA, left atrial; LVEF, left ventricular ejection fraction.

* *P* ≤ 0.05 vs. control.

Subsequently, we performed a correlation analysis between C1q and factors related to AF pathogenesis. Pearson/Spearman correlation analysis showed that plasma C1q concentration was negatively correlated with left atrial enlargement (*r* = −0.1975, *P* < 0.001), and showed weak negative correlations with LVEF (*r* = −0.075, *P* < 0.001) and creatinine (Cr) levels (*r* = −0.0595, *P* < 0.011). These results suggest that C1q may be closely associated with left atrial enlargement, ultimately contributing to the development of AF. However, further data is required to support this hypothesis (Table 5).

**Table 5:** Relationship between C1q and other variables.

Parameters	Correlation coefficient (*r*)	*p* value
Hypertension	−0.018	0.436
Diabetes	−0.035	0.135
BMI	0.024	0.306
SBP	0.023	0.319
Drinking	−0.030	0.195
Cr	−0.059	0.011*
LA enlargement	−0.198	0.001**
LVEF	−0.077	0.001**

BMI, body mass index; SBP, systolic blood pressure; Cr, creatinine; LA, left atrial; LVEF, left ventricular ejection fraction.

*p < 0.05, **p < 0.01 (two-sided tests).

### 3.3 Complex fractionated atrial electrograms(CFAE) in the superior wall of the left atrium

**Figure 4:**
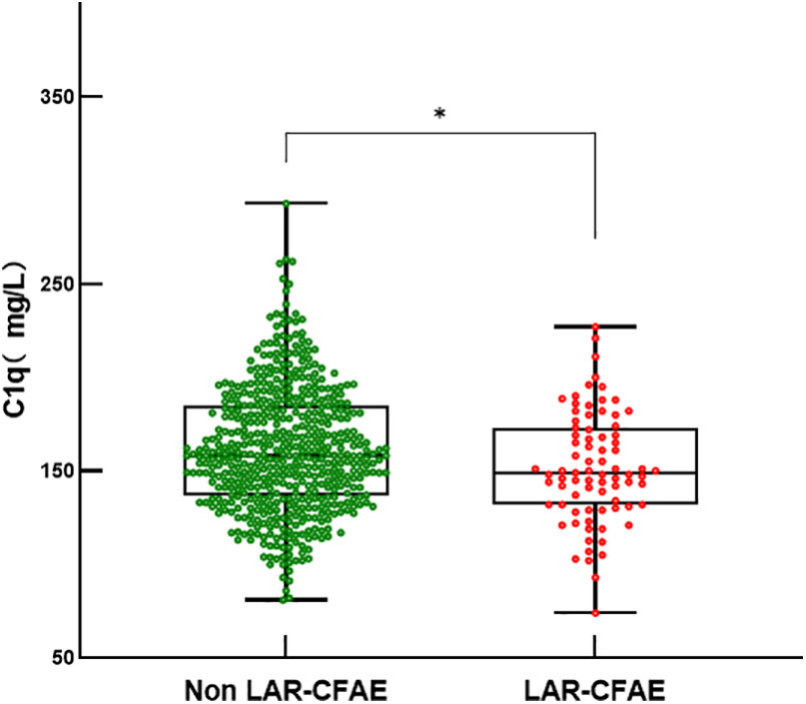
Serum C1q levels in fractionated atrial potentials and normal. Box and scatterplot of serum C 1q levels in patients of Fractionated Atrial Potentials and Normal groups; The horizontal line in the middle, top and bottom indicates the median value, the 75th and 25th percentiles, respectively; the dots represent each individual. “Ip < 0.01”. LAR, left atrial roof; CFAE, complex fractionated atrial electrograms.

Complex fractionated atrial electrograms (CFAE) in the superior left atrium are considered a major contributor to AF recurrence following pulmonary vein isolation. We analyzed intracardiac potential recordings from 600 patients with paroxysmal AF who underwent radiofrequency catheter ablation. Table 6 summarizes the clinical characteristics, laboratory findings, and imaging results of these patients. The results showed that 80 (13.3%) paroxysmal AF patients had CFAE in the superior left atrium and subsequently underwent additional radiofrequency ablation. We further divided the paroxysmal AF patients who underwent radiofrequency ablation into two groups: those with CFAE in the superior left atrium and those without. We found that C1q levels in patients with CFAE in the superior left atrium were significantly lower than in those without CFAE (151.81 ± 29.45 mg/L vs. 161.37 ± 33.13 mg/L, *P* = 0.015) (Table 6, Figure 4). To evaluate whether C1q is an independent risk factor for CFAE in the superior left atrium, we performed logistic regression analysis, including potential risk factors associated with CFAE. After adjusting for confounding factors, we found that plasma C1q levels (95% CI = 0.984–1.998, *P* = 0.031) and left atrial enlargement (95% CI = 1.514–81.119, *P* = 0.018) were independently associated with the presence of CFAE in paroxysmal AF patients. This indicates that C1q is an independent risk factor for the presence of CFAE in the superior left atrium of paroxysmal AF patients and is negatively correlated with the occurrence of CFAE. Interestingly, correlation analysis revealed that in paroxysmal AF patients with CFAE in the superior left atrium, C1q was positively correlated with female sex (*r* = 0.257, *P* < 0.001) but negatively correlated with left atrial enlargement (*r* = −0.293, *P* = 0.023) and diabetes (*r* = −0.085, *P* = 0.037) (Table 8). C1q showed no significant linear relationship with other parameters (*P* > 0.05).

**Table 6:** Clinical characteristics of AF patients that underwent AF catheter ablation (*n* = 600).

Parameters	Non LAR-CFAE	LAR-CFAE	*P*-value
	(*n* = 520)	(*n* = 80)	
Female *n* (%)	170 (32.7)	25 (31.3)	0.798
Age(years)	55.75 ± 11.18	55.52 ± 10.73	0.867
SBP (mmHg)	132.07 ± 16.32	130.01 ± 15.86	0.307
DBP (mmHg)	79.12 ± 11.30	81.28 ± 13.16	0.121
BMI (kg/m^2^)	26.12 ± 3.40	26.73 ± 3.08	0.864
Smoking	119 (22.9)	19 (23.6)	0.195
Drinking	131 (25.2)	18 (22.5)	0.605
Hypertension	240 (46.2)	35 (43.8)	0.688
Diabetes	102 (19.6)	19 (23.8)	0.392
CHA₂DS₂-VASc Score	2.97 ± 1.57	3.29 ± 1.82	0.418
Laboratory examination			
C1q(mg/L)	161.37 ± 33.13	158.81 ± 29.45	0.015
Cr (μmol/L)	75.70 ± 17.55	71.41 ± 16.81	0.041
Echocardiography			
LA enlargement	452 (86.7)	79 (98.7)	0.002
LVEF (%)	59.17 ± 4.76	58.66 ± 5.00	0.383

LAR, left atrial roof; SBP, systolic blood pressure; DBP, diastolic blood pressure; BMI, body mass index; Cr, creatinine; LA, left atrial; LA enlargement, High left atrium enlargement is defined as: left atrial area ≥20 cm^2^ or left atrial diameter >40 mm; LVEF, left ventricular ejection fraction.

**Table 8:** Paroxysmal AF: relationship between C1q and other variables.

Parameters	Correlation coefficient (r)	*p* value
Age	−0.005	0.907
Gender(female)	0.257	<0.001**
BMI	0.017	0.678
Hypertension	−0.038	0.359
Diabetes	−0.085	0.037*
Drinking	0.004	0.914
Cr	0.007	0.865
LA enlargement	−0.293	0.023*
LVEF	−0.005	0.897

BMI, body mass index; Cr, creatinine; LA, left atrial; LVEF, left ventricular ejection fraction.

*p < 0.05, **p < 0.01 (two-sided tests).

**Table 7:** LAR-CFAE: univariate and multivariable logistic regression analyses.

Parameters	Univariate analysis		Multivariable analysis	
	95% CI	*P*	95% CI	*P*
Complement C1q	0.983–0.998	0.016	0.984–0.998	0.031*
Age	0.977–1.020	0.867		
Female sex	0.564–1.554	0.798		
Hypertension	0.565–1.458	0.688		
Diabetes	0.730–2.231	0.392		
BMI	0.984–1.128	0.133		
SBP	0.978–1.007	0.306		
Drinking	0.492–1.511	0.604		
Smoking	0.603–1.827	0.864		
Left atrial enlargement	1.627–86.835	0.015	1.514–81.119	0.018*
LVEF	0.936–1.026	0.383		

LAR, left atrial roof; CFAE, complex fractionated atrial electrograms; BMI, body mass index; SBP, systolic blood pressure; LA, left atrial; LVEF, left ventricular ejection fraction.

* *P* ≤ 0.05 vs. control.

### 3.4 Complement C1q

**Figure 5:**
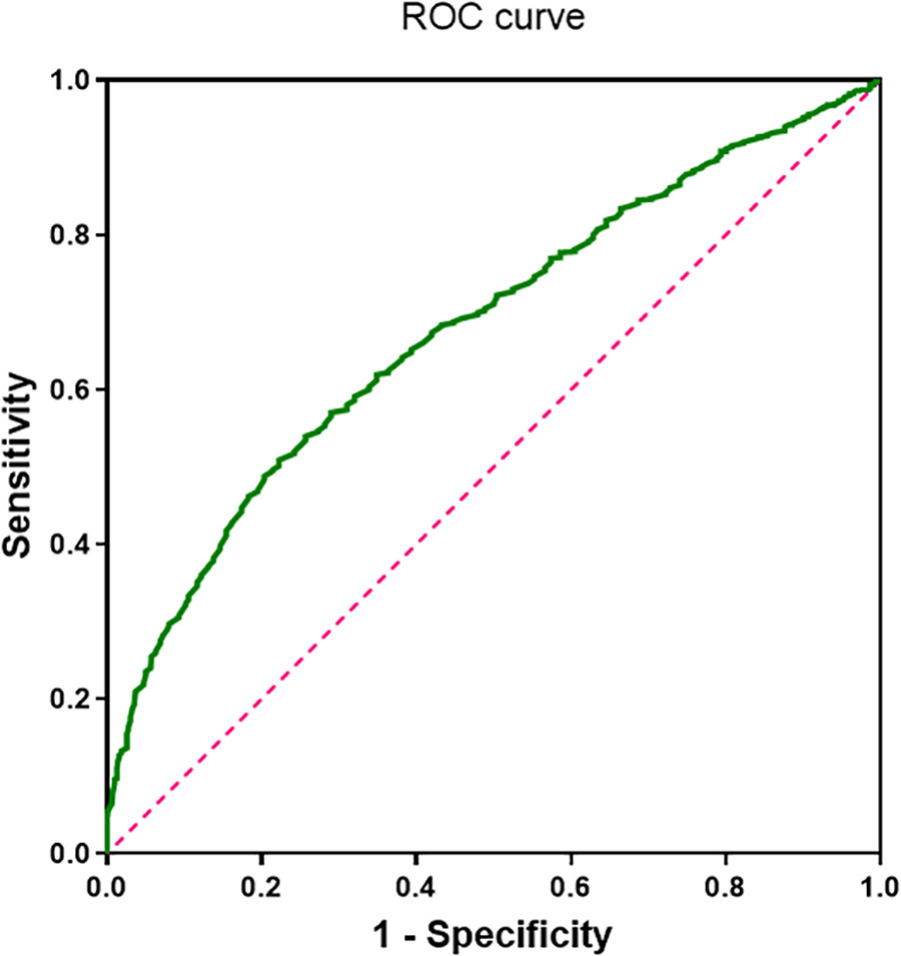
ROC curve of complement C1q in patients with atrial fibrillation (area under the curve = 0.68, 95% confidence interval 0.654–0.704; *P* < 0.001).

Our analysis indicates that complement C1q is an independent risk factor for AF and is associated with complex fractionated atrial electrograms (CFAE) in the superior left atrium of patients with paroxysmal AF. ROC curve analysis evaluated the predictive value of complement C1q for AF and determined an optimal cutoff value of 156.8 mg/L. The sensitivity and specificity of C1q in predicting AF were 0.51 and 0.77, respectively [area under the curve (AUC) = 0.679, 95% CI 0.654–0.704, *P* < 0.001; Figure 5], suggesting that complement C1q is a reliable risk predictor for AF.

**Figure 6:**
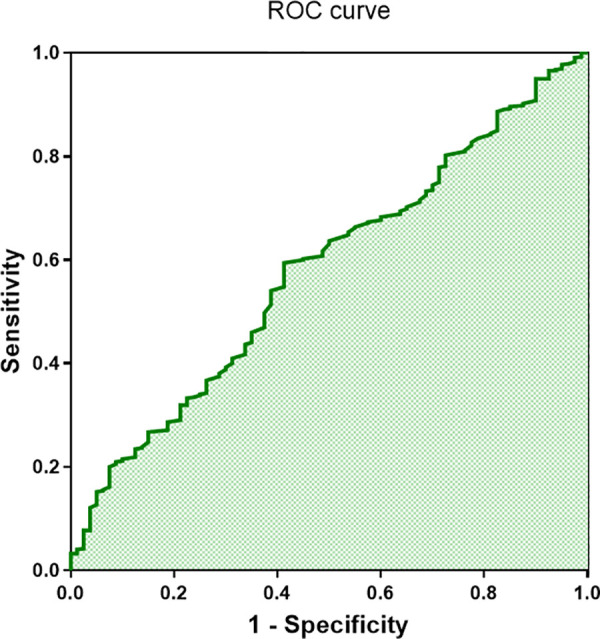
ROC curve of complement C1q in patients with complex fractionated atrial electrograms (CFAE) (area under the curve = 0.58, 95% confidence interval 0.515–0.643; *P* < 0.001).

Additionally, we performed ROC curve analysis using complement C1q as a potential predictor for CFAE in the superior left atrium of paroxysmal AF patients. The results indicated that C1q had a sensitivity of 0.59 and a specificity of 0.58 in predicting CFAE (AUC = 0.579, 95% CI 0.515–0.643, *P* = 0.022; Figure 6). This suggests that C1q has some predictive value for CFAE and may serve as a guiding indicator for comprehensive electrophysiological assessments during catheter ablation procedures in paroxysmal AF patients.

## 4 Discuss

Based on this retrospective case-control study, we report the following novel findings: plasma complement C1q levels were significantly downregulated in AF patients compared to non-AF controls, with more pronounced reductions observed in those with persistent AF. More importantly, further analysis revealed that decreased C1q levels were independently associated with complex fractionated atrial electrograms (CFAE) in the superior left atrium of paroxysmal AF patients undergoing catheter ablation. This suggests that the independent predictive role of C1q in AF may be linked to abnormal intra-atrial potentials, highlighting the need for a more comprehensive intra-atrial electrophysiological assessment during ablation procedures. Low complement levels are associated with atrial fibrillation compared to individuals without AF and may represent a potential underlying cause of impaired sinus rhythm maintenance following pulmonary vein isolation.

The development and progression of AF are influenced by multiple factors, including age, genetic predisposition, obesity, diabetes, hypertension, obstructive sleep apnea, valvular heart disease, cardiomyopathy, and heart failure (7). The complement system is a critical component of both specific and non-specific immune responses (20), capable of stimulating E-selectin expression and promoting the production of interleukins and other related inflammatory proteins through autocrine and paracrine mechanisms, thereby participating in the body's inflammatory response (21). As one of the key initiators of immune responses within the complement system, C1q deficiency has been closely associated with a shift from suppressive regulatory T cell (Treg) responses to pro-inflammatory Th17 responses (22). Some studies have suggested that inflammatory responses in the myocardium may serve as abnormal foci for AF initiation (23, 24), High-sensitivity C-reactive protein (hs-CRP) has also been identified as an independent predictor for the maintenance of sinus rhythm in AF patients (25). In recent years, C1q has been found to induce myocardial fibrosis and accelerate cardiomyocyte aging through the activation of inflammatory responses (26), the development of AF is driven by this key pathological change, which exacerbates atrial electrical disturbances. In this study, we found that serum complement C1q levels were significantly lower in AF patients compared to those with sinus rhythm, with even more pronounced reductions observed in patients with persistent AF. This suggests that complement C1q may be associated with AF pathogenesis. We further analyzed the correlation between C1q, left atrial enlargement, and left ventricular ejection fraction (LVEF). The results demonstrated that C1q was negatively correlated with left atrial enlargement and LVEF, suggesting that C1q may be significantly associated with impaired left atrial function, loss of myocardial fiber elasticity, and reduced overall left ventricular pumping function. Logistic regression analysis indicated that left atrial enlargement and C1q were independent risk factors for AF, further supporting this hypothesis. AF, one of the most common arrhythmias in clinical practice, not only reduces cardiac pumping efficiency but also increases the risk of stroke and heart failure, significantly impairing patients' quality of life. We identified a potential abnormality in C1q levels among AF patients. ROC curve analysis demonstrated good sensitivity and specificity of C1q for predicting AF. Furthermore, when the ROC cutoff value was set at 156.80 mg/L, C1q exhibited strong specificity for predicting AF. Patients with C1q levels below this threshold were more frequently identified as having persistent AF. Therefore, serum C1q may serve as a biomarker for AF and play an important role in AF screening and management.

CFAE in the superior left atrium is a common cause of AF recurrence following pulmonary vein isolation therapy. CFAE manifests as fragmented or fractionated electrical signals during cardiac electrophysiological examination and is associated with atrial structural remodeling, local inflammation, and the degree of fibrosis. CFAE ablation has been shown to modify the atrial substrate that sustains AF, leading to favorable long-term outcomes in patients with paroxysmal AF (27–29). Interestingly, we found that C1q was significantly associated with CFAE in the superior left atrium of patients with paroxysmal AF. Logistic regression analysis indicated that C1q is an independent risk factor for superior left atrial CFAE in paroxysmal AF patients and is also correlated with left atrial enlargement, sex, and history of diabetes. Therefore, we further investigated the predictive role of C1q for superior left atrial CFAE in paroxysmal AF patients. ROC curve analysis demonstrated that C1q had good predictive value for CFAE in these patients. This suggests that C1q may serve as an independent risk predictor for detecting potential CFAE during catheter ablation procedures in patients with paroxysmal AF. CFAE has been shown to induce and exacerbate AF episodes and is even associated with AF recurrence following catheter-based radiofrequency ablation (30). This finding further supports our hypothesis that C1q is an independent risk factor for AF and has strong predictive value for the condition.

Complement C1q is closely associated with various cardiovascular diseases. Complement C1q has been shown to increase the potential risk of adolescent obesity and metabolic syndrome (Mets) by modulating obesity-related pathogenic factors (31), It can also reduce the clearance of myocardial metabolites by mediating alcohol-induced hepatocyte injury (32), thereby exacerbating chronic myocardial damage. C1q has been proven to induce changes in LDL levels and can serve as a predictor of coronary atherosclerosis (33). Furthermore, C1q can amplify hypertension-related risk factors to a certain extent, leading to abnormal fluctuations in blood pressure levels and increased myocardial load (34). Other studies have found that C1q is involved in the early activation of fibroblasts and type I alveolar epithelial cells and participates in the progression of pulmonary fibrosis by modulating signaling pathways (35). It can further deteriorate lung function in smokers (36), ultimately leading to increased cardiac load and myocardial fibrosis. These factors may serve as potential triggers for increased myocardial load and the development of myocardial fibrosis. In addition, C1q has many other important functions. For example, C1q has been shown to enhance neuronal viability and reduce neuronal damage by regulating nerve growth factor (NGF) and neurotrophin-3 (NT-3) (37); In terms of tumor survival, C1q participates in cancer cell proliferation and tumor-associated angiogenesis by protecting against oxidative stress-mediated apoptosis in the tumor microenvironment (38); In autoimmune responses, C1q controls and modulates innate and adaptive immune pathways through complement-independent mechanisms, contributing to the development of early-onset systemic lupus erythematosus and preventing opportunistic bacterial infections (39).

The potential relationship between complement C1q and thrombosis remains unclear. Interestingly, while exploring the relationship between AF and complement C1q, we found that C1q may have a potential link to intra-atrial thrombus formation in AF patients. It is well known that a thrombus is a blood clot composed of platelets, coagulation factors, fibrin, and sometimes red and white blood cells. Intra-atrial thrombus formation is a major contributor to severe complications in AF patients. Studies have found that C1q exhibits high affinity for von Willebrand factor (vWF) during the coagulation process and participates in platelet adhesion (40). C1q deficiency or abnormal depletion leads to an increased platelet count and significantly shortened activated partial thromboplastin time (APTT) (41–44), which undoubtedly elevates the risk of thrombus formation. Moreover, cardiac fibroblasts have been found to be involved in the expression of tissue factor (30). In non-valvular AF patients, tissue factor expression in the atrial endothelium has been shown to be potentially associated with intra-atrial thrombus formation (35), C1q is involved in the activation of downstream factors of tissue factor and the initiation of TF-related coagulation responses (36), suggesting that C1q may contribute to intra-atrial thrombus formation by participating in tissue factor-related coagulation pathways. More importantly, a reduction in tissue factor levels within a certain range can downregulate uPA expression and inhibit its activity in myocardial fibrosis. This trend is particularly prominent in women but shows the opposite tendency in men (45). The extensive consumption of tissue factor and other coagulation factors during thrombus formation may lead to a decrease in uPA, thereby promoting thrombus formation. This mechanism could be related to the higher risk of thrombus formation observed in female patients compared to males. Therefore, we hypothesize that complement C1q may be involved in the essential processes of thrombus formation and may play a more active role in the atrium. However, further experimental validation is required to support this hypothesis.

In conclusion, our study suggests that complement C1q may serve as a novel biomarker for AF. Reduced C1q levels in AF patients may indicate chronic inflammatory changes in atrial tissue, the presence of CFAE, and could even increase the potential risk of intra-atrial thrombus formation.

Therefore, plasma C1q levels may have potential value in the diagnosis of AF in the general population and could guide more detailed electrophysiological examinations during catheter ablation procedures. Low complement levels are associated with atrial fibrillation compared to individuals without AF. Combining C1q levels with other clinical indicators may help predict the risk of AF onset and guide improvements in surgical strategies, providing new avenues for the clinical assessment and treatment of AF patients. However, this study has several limitations. First, our findings derived exclusively from AF patients who ultimately underwent catheter ablation for diverse clinical indications (e.g., symptomatic or drug-refractory AF) may not fully represent the broader AF population, particularly those managed medically or with watchful waiting, potentially introducing selection bias. Furthermore, we did not conduct follow-up with paroxysmal AF patients who underwent radiofrequency ablation, preventing further investigation into the relationship between complement C1q, superior left atrial CFAE, and postoperative AF recurrence. Therefore, cohort studies encompassing broader AF populations remain necessary to validate these findings. We have initiated plans to establish a prospective multicenter registry aimed at further verifying this association while longitudinally assessing the complement system's potential impact on AF ablation outcomes.

## Data Availability

The raw data supporting the conclusions of this article will be made available by the authors, without undue reservation.
